# Modeling and Synthesis of Ag and Ag/Ni Allied Bimetallic Nanoparticles by Green Method: Optical and Biological Properties

**DOI:** 10.1155/2018/9658080

**Published:** 2018-01-01

**Authors:** Anuoluwa Abimbola Akinsiku, Enock Olugbenga Dare, Kolawole Oluseyi Ajanaku, Olayinka Oyewale Ajani, Joseph Adebisi O. Olugbuyiro, Tolutope Oluwasegun Siyanbola, Oluwaseun Ejilude, Moses Eterigho Emetere

**Affiliations:** ^1^Department of Chemistry, Covenant University, PMB 1023, Ota, Ogun State, Nigeria; ^2^Department of Chemistry, Federal University of Agriculture, PMB 2240, Alabata Road, Abeokuta, Nigeria; ^3^Department of Medical and Parasitology, Sacred Heart Hospitals, Lantoro, Abeokuta, Nigeria; ^4^Department of Physics, Covenant University, PMB 1023, Ota, Ogun State, Nigeria; ^5^Department of Mechanical Engineering Science, University of Johannesburg, Auckland Park Kingsway Campus, Johannesburg 2006, South Africa

## Abstract

In the quest for environmental remediation which involves eco-friendly synthetic routes, we herein report synthesis and modeling of silver nanoparticles (Ag NPs) and silver/nickel allied bimetallic nanoparticles (Ag/Ni NPs) using plant-extract reduction method. Secondary metabolites in the leaf extract of* Canna indica* acted as reducing agent. Electronic transitions resulted in emergence of surface plasmon resonance in the regions of 416 nm (Ag NPs) and 421 nm (Ag/Ni NPs) during optical measurements. Further characterizations were done using TEM and EDX. Antimicrobial activity of the nanoparticles against clinical isolates was highly significant as *P* < 0.05. These findings suggest application of Ag NPs as antibacterial agent against* E. coli, S. pyogenes,* and antifungal agent against* C. albicans*. Possible antibacterial drugs against* S. pyogenes* and* E. coli* can also be designed using Ag/Ni nanohybrid based on their strong inhibition activities. Similarly, the enhanced SPR in the nanoparticles is suggested for applications in optical materials, as good absorbers and scatters of visible light. Theoretical model clarified that the experiment observation on the relationship between metallic nanoparticles penetration through peptidoglycan layers and the activeness of microbial species depends on the nature of the nanoparticles and pore size of the layer.

## 1. Introduction

Recently, paradigm shift in technology has led to the synthetic protocols involving application of green chemistry, part of environmental remediation, encompassing use of biomaterials because of their eco-friendliness. The use of biological materials as sensors, information storage devices, and bimolecular array is on the increase. No doubt, novel characteristics are possessed by materials on nanometre scale, as this creates special interest which is applicable virtually to every aspect of life including medicine, agriculture, and polymer industry among others [1]. There is a growing interest in magnetic NPs of nickel origin due to their superior magnetic characteristics which has a useful application in medicine (as magnetic drug delivery) and therapeutics [2–4]. Imran Din and Rani [5] reviewed progress in the “green” protocols for the syntheses and stabilization of nickel and nickel oxide nanoparticles when* Azadirachta indica *and* Psidium guajava* leaves were utilized in the synthesis of NiO and Ni nanoparticles with an average size of 17−77 nm. Face-centered cubic Ni NPs were reported by Chen et al. in which Ni (NO_3_)_2_ was reduced with* Medicago sativa *(alfalfa) extract. Ni NPs were also synthesized by Chen et al. with the leaf extract of* Ocimum sanctum *as reducing and stabilizing agents: hydrated electrons of* O. sanctum *aqueous leaf extract were considered to have reduced Ni(II) ions into Ni(0) [6, 7].

Biosynthesized conjugated bimetallic nanoparticles are now used in biomedical field, imaging, luminescence tagging, labeling, and drug delivery due to their compatibility in* in vivo* screening [8]. Glucose-capped nickel nanoparticles (G-Ni NPs) were synthesized by Vaseem et al. via aqueous solution method in which glucose bifunctioned as capping agent and a reducing agent [9].* Canna indica* (Linn.), commonly known as Indian shot belonging to the family Cannaceae is a medicinal plant of diverse uses. The herb consists of rhizomatous root stocks; reddish or yellowish showy flowers, which encompass a variable number of rounds; and shiny black seeds. In folkloric medicine, root decoction is used for the treatment of fever, dropsy, and dyspepsia [10]. Seed juice is used to relieve ear aches. Many varieties of* C. indica *are grown in the gardens and around houses for beautification. Ethnomedical use of* C. indica* leaves include antimicrobial, analgesic, and anthelmintic activities [11]. Research has shown that extraction of* C. indica* rhizomes using water was effective for HIV-1 reverse transcriptase inhibitory activity [12].

From survey, biological syntheses of nanomaterials make use of bacteria, yeasts, fungi, and algae (microorganisms). The use of plants or plant extracts for metal and metal hybrid nanoparticles synthesis is currently a new research focus that has gained wide acceptance [13]. As the demands for commercial nanoparticles are on the increase due to their wider applications in many fields, plant mediated green synthesis presents a cost-effective alternative method that is eco-friendly and sustainable [14]. The technique provides stable nanoparticles dispersions that resist aggregation in biological media and have high resistance to oxidation which is of significant importance [15]. Consequently, there is a need to solve public health problem of drug resistance by the disease causing microbes; new drug procedure needs to be adopted. It is noteworthy that Nigeria is endowed with natural biodiversity whose potential for developing novel health care and active drug candidates through green method has been underutilized. Plant extract with adequate phytochemicals has been confirmed to be faster in initiating bioreduction compared to microbes and the conventional chemical methods [16].

Quite a number of literatures have reported syntheses of allied silver-nickel nanoparticles using various chemical methods. Adekoya et al. [17] synthesized optically active fractal seed mediated silver-nickel bimetallic nanoparticles by chemical method; nevertheless, no investigations were carried out using* Canna indica* green-mediated route technique for the synthesis of Ag/Ni bimetallic nanoparticles. In view of the potential of Ag and Ni NPs having good antibacterial activity against some pathogenic organisms [18] and their optical properties as a result of shift of surface plasmon resonance wavelength (*λ*_SPR_) to a longer wavelength based on particle size [19], we report facile green synthetic methodology and theoretical modeling of silver and silver/nickel hybrid nanoparticles that entails in situ reduction of aqueous Ag(I) and Ni(II) ions. The phytochemicals present in the aqueous leaf extract of* Canna indica* were considered to act as reducing/capping agents. UV-Visible spectrophotometer was used to monitor the optical properties; morphological characterization, size determination, elemental analysis, and antimicrobial screening of the biosynthesized nanoparticles were also carried out.

## 2. Experimental Details

### 2.1. Materials

*Canna indica *leaf extract (Indian shot), Whatman number 1 filter paper, distilled deionized water, silver nitrate (AgNO_3_), and nickel nitrate hexahydrate Ni(NO_3_)_2_·6H_2_O, commercially obtained from Sigma-Aldrich Company, UK, were used as obtained.

#### 2.1.1. Test Microorganisms

Freshly cultured clinical isolates of* Escherichia coli*,* Pseudomonas aeruginosa* (gram-negative bacteria),* Staphylococcus aureus*,* Streptococcus pyogenes *(gram-positive bacteria),* Candida albicans,* and* Trichophyton rubrum *(Fungi) were collected from the Department of Medical Microbiology and Parasitology, Sacred Heart Hospital, Lantoro, Abeokuta, in Nigeria.

#### 2.1.2. Preparation of Leaf Extract

Indian shot plant was collected from a garden at Atan-Iju, Ogun State, Nigeria. Plant identification and authentication were carried out at Forest Research Institute of Nigeria (FRIN); voucher specimen FHI 109928 was deposited at the herbarium headquarters, Ibadan, Nigeria. Fresh leafy part of the plant was washed with distilled water, finely cut, and ground using mortar and pestle. It was then extracted at a ratio of 1 : 5 wt/v using distilled deionized water, filtered with Whatman number 1 filter paper, and then kept at 4°C. The filtrate was used for phytochemical screening and nanoparticle synthesis. The procedure is modified from previous work [20].

#### 2.1.3. Phytochemical Screening of the Plant Extracts

Plant extract was screened to identify the phytochemicals present according to literature [21].

#### 2.1.4. Syntheses of Ag and Ag/Ni Bimetallic Nanoparticles

Metallic nanoparticles were prepared by plant-extract reduction method with modification to previous work [22]. For the synthesis of Ag NPs, 10 mL of the 0.2 g/mL aqueous filtrate of* C. indica *extract was added to 100 mL of varied concentrations of aqueous silver nitrate solution (0.5–2.0 mM). The reaction mixture was continuously stirred and gradually heated to 70°C on a hotplate. In the case of Ag/Ni bimetallic nanoparticle synthesis, 20 mL of the plant extract was added to equal molar concentration mixture of 100 mL AgNO_3_ and 100 mL Ni(NO_3_)_2_·6H_2_O in a beaker. Precursor concentrations were varied between 0.5 and 3.0 mM. Initial colour of the mixture was noted. The resulting mixture was continuously stirred and gradually heated to 70°C until there was a change in colour. Bioreductions of Ag(I) ions to Ags(0) and Ni(II) to Ni(0) were monitored by taking samples at varied time intervals, using UV-Vis spectrophotometer (double beam Thermo Scientific GENESYS 10S model), starting from the 5th minute until a noticeable colour change and appearance of surface plasmon resonance band (SPRB). Sample was placed in quartz cuvette, operated at a resolution of 1 nm so as to measure the absorbance.

#### 2.1.5. Isolation of Metallic Ag and Ag/Ni Nanoparticles

The biosynthesized nanoparticles were collected by centrifugation using centrifuge model 0508-1, operated at 5000 rpm for 30 minutes. For purification, the nanoparticles suspension was redispersed in distilled deionized water so as to remove the unbounded organics and finally centrifuged at 5,000 rpm for 10 minutes. The suspension was oven dried and kept in Eppendorf tubes for further characterizations.

### 2.2. Characterization

#### 2.2.1. Optical Characterization

Optical properties of the prepared metallic nanoparticles were determined using a double beam Thermo Scientific GENESYS 10S UV-Vis spectrophotometer between 200 and 800 nm wavelength ranges. Absorbance measurement was carried out by placing each aliquot sample taken at time intervals in quartz cuvette (1 cm path length), operated at a resolution of 1 nm, using distilled deionized water as blank.

#### 2.2.2. Structural Characterization

Structural, morphological characteristics and size determination of the particles were verified with Technai G2 transmission electron microscope (TEM) coupled with an energy-dispersive X-ray spectrometer (EDX), operated at an accelerating voltage of 200 KeV and 20 *μ*A current. Samples for TEM analysis were prepared by drop-coating Ag and Ag/Ni suspensions onto carbon-coated copper TEM grids. The films on the TEM grids were allowed to dry prior to measurement.

### 2.3. Antimicrobial Activity

#### 2.3.1. Turbidity Standard for Inocula Preparation

McFarland standard on laboratory guidance was used for the standardization of organisms for susceptibility testing, using a modified method by British Society for Antimicrobial Chemotherapy. BaSO_4_ turbidity standard equivalent to 0.5 McFarland standards or its optical equivalent was used. The 0.5 McFarland standard was prepared by adding 0.5 mL of 0.048 M BaCl_2_ of (1.175% w/v) BaCl_2_ in 2H_2_O to 99.5 mL of 0.18 M H_2_SO_4_ with constant stirring to maintain a suspension. The correct density of the turbidity standard was verified by a pg instrument UV-Vis spectrophotometer model T90+, with 1 cm light path, and matched cuvette to determine the optical density at a wavelength of 625 nm. The acceptable range for the standard is 0.08–0.13 for 0.5 McFarland standard which is equivalent to 1.5 × 10^8^ bacterial cells per mL. The standard was distributed into screw cap tubes of the same size and volume, similar to method of growing or diluting the bacterial inocula. The tubes were tightly sealed to prevent loss by evaporation. They were then stored in the dark at room temperature. The turbidity standard was vigorously agitated on a vortex mixer before use. The standard remains potent for six months; appearance of large particles in the standard is an indication of expiration [23].

#### 2.3.2. Preparation of Inocula

The microbial strains were propagated in Mueller Hinton broth, prepared by dispersing 5 mL of the prepared broth medium into each screw capped test tube, sterilized by autoclaving at 12°C for 15 minutes. The test tubes were cooled and kept in an incubator for 24 hours at 37°C in order to determine the sterility. The isolates were inoculated into the sterilized test tubes containing the medium and placed in an incubator overnight at 37°C. Appearance of turbidity in broth culture was adjusted equivalent to 0.5 McFarland standards. This was done to obtain standardized suspension. Sterile normal saline was added in order to obtain turbidity optically comparable to that of the 0.5 McFarland standards or against a white background with contrasting black line. The McFarland 0.5 standard provided turbidity comparable to bacterial suspension containing 1.5 × 10^8^ cfu/mL [24]. The suspension was used within 5 minutes so as to avoid population increase.

#### 2.3.3. Sensitivity of Test Organisms

Antimicrobial properties of the biosynthesized nanoparticles were investigated in the form of sensitivity testing, using modified version of the method described by Aida [25]. The test organisms were collected on sterile agar slant and incubated at 37°C for 24 hours. The following biochemical analyses were carried out on the bacteria test organisms: sugar fermentation, citrate utilization, oxidase reaction, Voges-Proskauer, methyl red, capsule staining, spore staining, motility, indole test, urease test, hydrogen sulphide test, gelatin liquefaction, and gram staining. Conversely, the fungus* Candida albicans* was identified by gram staining, germ tube test, sugar fermentation, and assimilation tests.* Trichophyton rubrum* (fungus) was identified macroscopically and microscopically using lactophenol cotton blue stains. These were then kept as stock culture on slant in the refrigerator at 4°C. The procedure was in agreement with recommended standards of National Committee for Clinical Laboratory Standards (NCCLS) [24].

#### 2.3.4. Agar Well Diffusion Method

Antibacterial activity of synthesized nanoparticles was evaluated by the well plate agar diffusion method as described in the Aida modified method [25]. The microbial cultures were adjusted to 0.5 McFarland turbidity standards and inoculated on Muller-Hinton agar plate of diameter 9 cm. The plate was flooded with each of the standardized test organisms (1 mL) and then swirled. Excess inoculum was carefully decanted. A sterile cork borer was used to make wells (6 mm in diameter) on the agar plates. Aliquots of the nanoparticle dilutions (0.1 mL) were reconstituted in 50% DMSO at concentrations of 100 mg/mL and applied on each of the wells in the culture plates previously inoculated with the test organisms. However, each extract was tested in duplicate with 0.1 mL of 5 *μ*g/mL ciprofloxacin as positive control for bacteria and fluconazole as positive control for fungi. These were then left on the bench for 1 hour for proper diffusion of the nanoparticles [24]. Thereafter, the plates were incubated at 37°C for 24 hours for bacteria and yeast and at 28°C for 72 hours for* T. rubrum*. Antimicrobial activity was determined by measuring the zone of inhibition around each well (excluding the diameter of the well) for nanoparticles obtained from the plant extract. Duplicate tests were conducted against each organism and significant growth inhibitions were found using analysis of variance (ANOVA), SPSS statistical tool.

#### 2.3.5. Minimum Inhibitory Concentration (MIC)

Serial dilution method was employed according to CLSI guidelines. Sterile test tubes (12) were arranged in a rack. 1 mL of sterile nutrient broth was added to tube labeled 2 to 10. 1 mL of known nutrients broth concentration was added to tubes 1 and 2. Afterwards, serial doubling dilution from tube 2 to tube 10 was made, while the remaining 1 mL was discarded. 1 mL of ciprofloxacin was added to tube 11 (positive control) and water to tube 12 (negative control). 1 mL of 0.5 McFarland was added overnight and broth culture to all the tubes and then covered. The experiment was incubated overnight at 37°C and observed for the highest dilution showing no turbidity. The zone of inhibition was then verified and interpreted according to CLSI guidelines [26]; the MIC was determined.

#### 2.3.6. Minimum Bactericidal Concentration (MBC) and Minimum Fungicidal Concentration (MFC)

MBC, the lowest concentration of antibiotic agent that kills at least 99.9% of the organisms, was determined by using Doughari et al. method. 0.5 mL of the sample was removed from those tubes from MIC which did not show any visible sign of growth and inoculated on sterile Mueller Hinton agar by streaking. The plates were then incubated at 37°C for 24 hours. The concentration at which no visible growth was seen was recorded as the minimum bactericidal concentration (MBC). For MFC, 0.5 mL of the sample which showed no visible sign of growth during MIC screening was taken from the test tubes and then inoculated on sterile potato dextrose agar by streaking. The plates were then incubated at 37°C for 24 hours. The concentration at which no visible growth was seen was recorded as the minimum fungicidal concentration [27].

## 3. Results and Discussion

### 3.1. Optical Properties of the Metallic Nanoparticles

**Figure 1:**
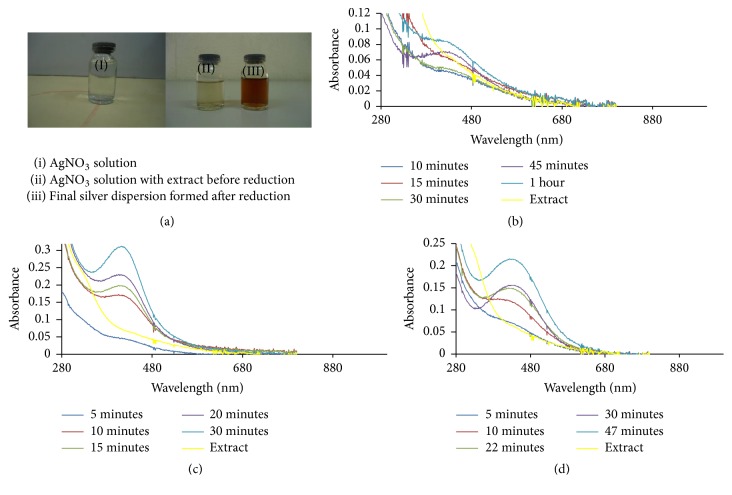
(a) Colour dispersion before and after nanoparticles formation, UV-Vis spectra of Ag NPs prepared by reducing (b) 0.5 mM, (c) 1.0 mM, and (d) 2.0 mM precursor solutions using the extract of* C. indica* at 70°C.

**Figure 2 fig2:**
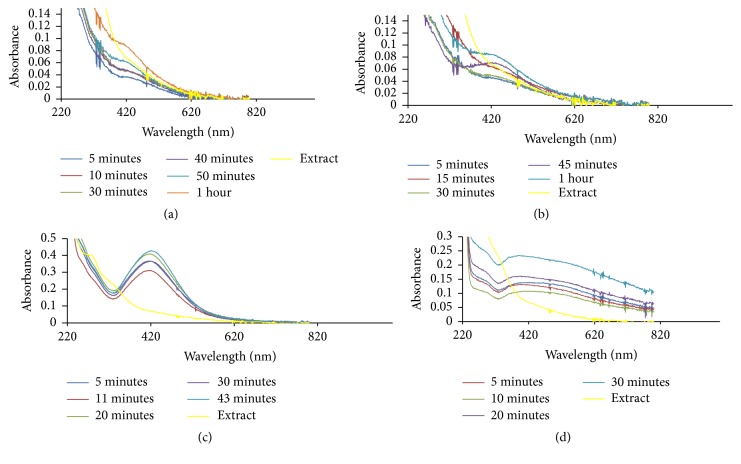
UV-Vis spectra of Ag/Ni bimetallic nanoparticles prepared by reducing (a) 0.5 mM, (b) 1.0 mM, (c) 2.0 mM, and (d) 3.0 mM solutions using the extract of* C. indica* leaves at 70°C. The blue curve represents surface plasmon resonance after 30 minutes.

**Figure 3:**
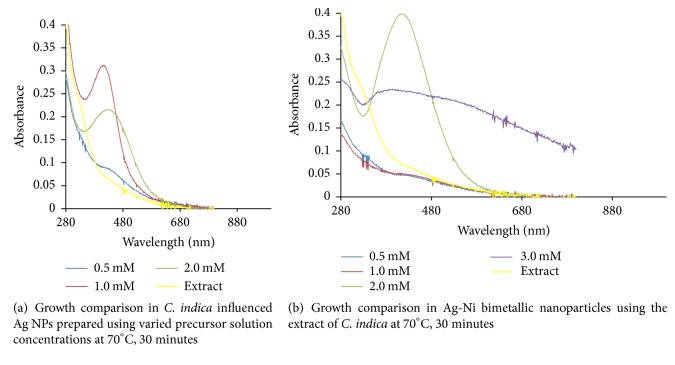


UV/Visible spectra of the biosynthesized silver nanoparticles (Ag NPs) and silver-nickel (Ag/Ni) bimetallic nanoparticles (Ag/Ni NPs) as a result of photon absorption by their solutions are displayed in Figures 1 [Fig fig2]–3. There was a noticeable colour change from light brown to deep brown which signalled formation of nanoparticles (Figure 1(a)). This is as a result of electronic transitions within the structures of Ag and Ag/Ni nanoclusters as they interacted with light. The electronic transitions within the structures of metallic nanoparticles resulted in emergence of surface plasmon resonance (SPR) which increased in peak intensity and confirmed Ag and Ag/Ni NPs formation [28]; bioreduction of Ag^+^ to Ag^0^ is an indication for potential application as excellent absorbers of visible light scatter and absorbers.

Ag NPs formation had maximum absorption in the visible region with absorption wavelength of 416 nm and maximum intensity of 0.312 a.u. There was electron confinement effect when 1.0 mM AgNO_3_ was reduced, and this culminated in sharp peaks and strong intensity observed. Intensity of absorption in the hybrid nanoparticles prepared from 2.0 mM precursor solution was in contrast to the maximum intensity of absorption noticed in the corresponding monometallic Ag NPs. In the bimetallic Ag/Ni NPs, there were narrow absorption spectra which increased in peak intensity without any shift in wavelength (421 nm). Hence, this signified presence of spherically shaped nanoparticles. Moreover, surface of the hybrid nanoparticles in Figure 2(c) is proposed to be enriched with silver, optically enhanced by nickel which is in line with previous work [29]. The observed narrow peak also depicted confinement of excitons in the nanoparticles as shown in Figures 1(c) and 2(c). Broad band in absorption wavelength between 400 and 450 nm in other nanobimetallic solutions of 0.5, 1.0, and 3.0 mM precursor mixture suggested aggregation and polydispersed structures [30] and this could be as a result of interaction between solute and solvent, hereby reducing the structural resolution and maximum energy of the reaction. The observed spectra overlap at 20th and 30th minute with no further changes in intensity indicating reaction completion. In Ag/Ni hybrid nanoparticles formation, an unprecedented bioreduction feature was observed, resulting in nucleation and growth of the nanoparticles within 5 minutes of reaction in all the concentrations. This observation was different in the case of monometallic Ag NPs in which nucleation and formation were delayed till 20 minutes in 0.5 mM metal precursor but was relatively faster at higher precursor solution concentrations (1.0 and 2.0 mM). The belated onset growth and reduction of 0.5 mM precursor suggested diverse mechanistic character in the formation of nanoparticles [31].

However, presence of nickel (Ni) in the hybrid synthesis of course led to a red shift in the absorbance wavelength from 416.0 to 421 nm, as observed in the reduced 2.0 mM precursor solution of Ag/Ni NPs. There was an obvious increase in intensity of absorption when compared with the corresponding Ag NPs. Growth comparison and optimum concentration for Ag NPs and Ag/Ni bimetallic synthesized at 70°C are displayed in Figures 3(a) and 3(b), respectively. Size increase due to red shift of absorption wavelength with an enhanced surface plasmon resonance was noted for application in biodiagnostic, optical materials, optoelectronics, and good absorbers of visible light and scatters [32].

**Scheme 1:**
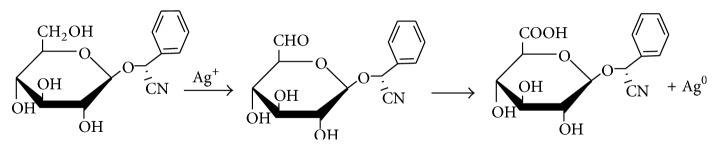
Bioreduction of silver ion to silver nanoparticles by glycosides.

**Scheme 2:**
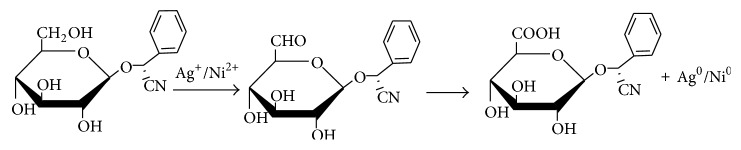
Bioreduction of silver/nickel ions to silver/nickel nanoparticles by glycosides.

**Scheme 3:**
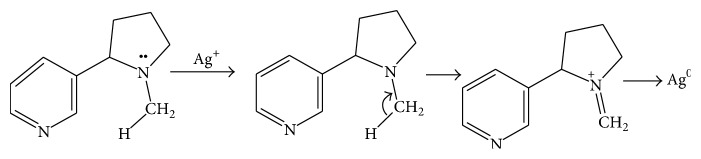
Bioreduction of silver ion to silver nanoparticles by alkaloids.

**Scheme 4:**
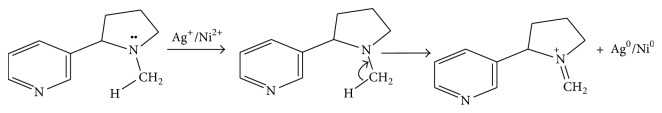
Bioreduction of silver/nickel ions to silver/nickel nanoparticles by alkaloids.

**Scheme 5:**
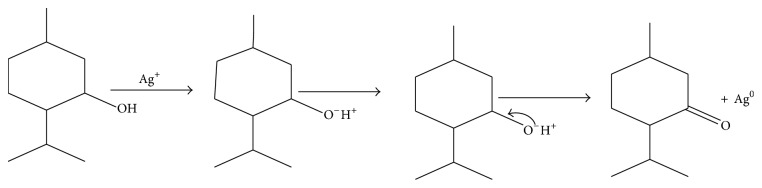
Bioreduction of silver ion to silver nanoparticles by terpenoids.

**Scheme 6:**
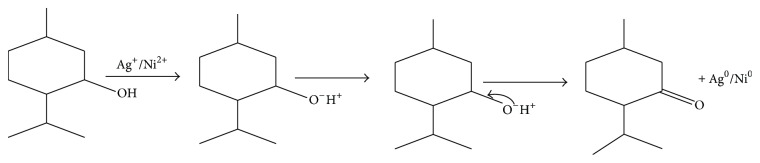
Bioreduction of silver/nickel ions to silver/nickel nanoparticles by terpenoids.

Biomolecules which acted as the reducing and capping/stabilizing agents for the newly formed nanoparticles were considered to be adequate as a result of unprecedented fast and successful bioreduction [33]. It is noteworthy that* Canna indica *leaf extracts contained the following secondary metabolite: alkaloids, glycosides, and terpenoids were identified in the water extract (Table 1). Tannins, flavonoids, and saponins were also detected in addition when leaf part of the plant was extracted with methanol (Table 1). These were confirmed thorough phytochemical screening. Despite choice of water as the extraction solvent (“green” part of the study) with limited amount of phytochemicals observed compared with methanol extract, the bioreduction and nanoparticles formation were successful. Proposed mechanisms of nanoparticles formation are presented in Schemes 1–6.

### 3.2. Proposed Mechanisms of Reactions

 See Schemes 1–6.

### 3.3. Morphology of the Metallic Nanoparticles

**Figure 4:**
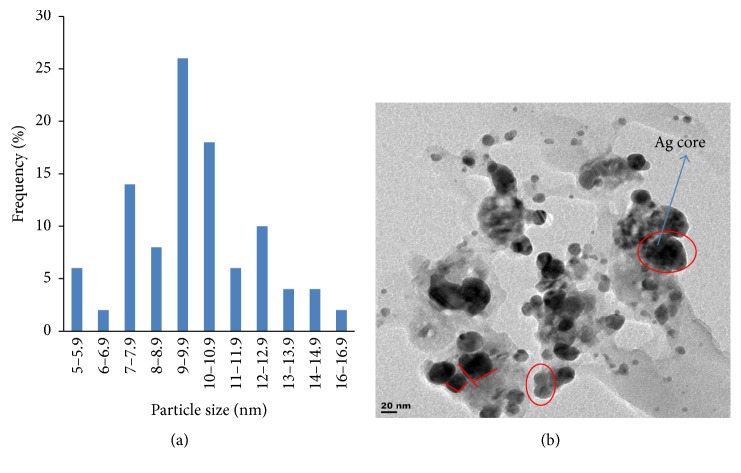
(a) Particle size distribution histogram of Ag/Ni determined from TEM images. (b) Representative TEM image of the bimetallic Ag/Ni NPs under* C. indica* influenced synthesis.

**Figure 5:**
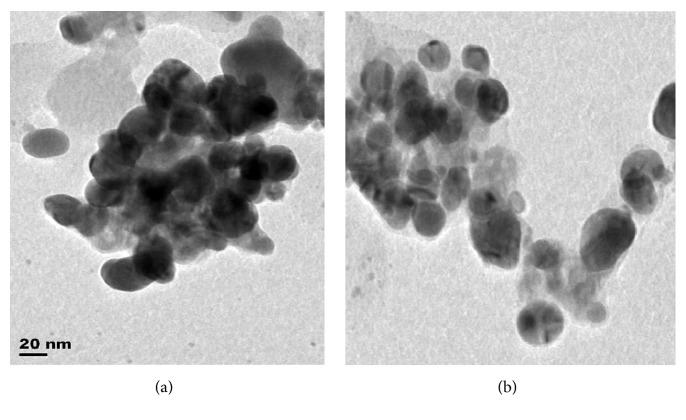
Representative TEM images of Ag/Ni bimetallic NPs derived from* C. indicia *leaf extract.

**Figure 6:**
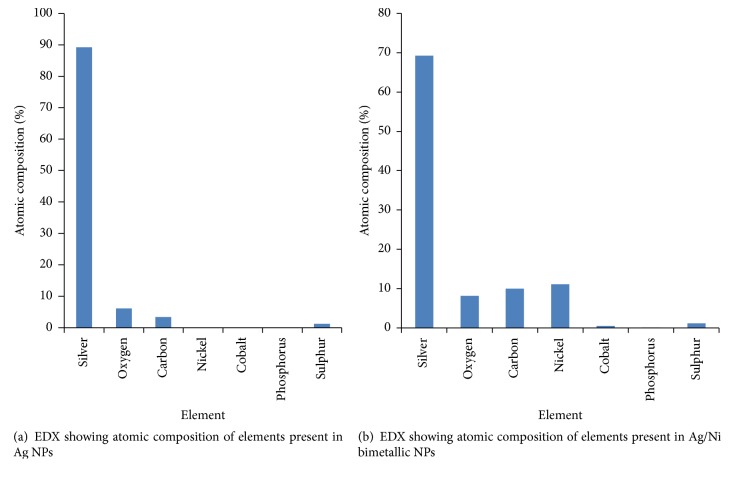


Particle size distribution histogram and TEM image of the biosynthesized Ag/Ni bimetallic nanoparticles are depicted in Figures 4(a) and 4(b), respectively. Other representative TEM images are shown in Figures 5(a) and 5(b). TEM image revealed quasi-spherical shapes of average diameter of 9.10 ± 1.12 nm for Ag NPs, while micrograph of the bimetallic Ag/Ni NPs revealed different shapes: cube with truncated/irregular edges plausibly due to the effect of Ostwald ripening and multiply twinned hybrid after 30 minutes of the reaction with a mean particle size of 9.86 ± 2.37 nm [34]. Structural elucidation from TEM image also revealed formation of core-shell Ag/Ni nanoparticles. The denser silver particles were distinctly visible in the TEM image. The Ag nanoparticles appeared as a dark core with Ni particles appearing less dark on the surface (Figure 2(d)). EDX analysis showing elemental compositions confirmed presence of nickel in the nanohybrid which was silver/nickel enriched with organic capping agent of carbon content which originated from plant extract (Figure 6(b)).

This finding is unique in biosynthesis of nanoparticles. Related finding was reported by Mntungwa et al. [35]; nevertheless, chemical method was applied to obtain the core-shell structure. Mechanism of the process could be explained as the ability of the plant extract to reduce the metal ions, followed by nucleation of metal atoms. Ostwald's ripening took place as a result of redissolution of high solubility and surface energy of smaller particles in the solution as the growth of larger particles continued more, as described by Lifshic and Slezov [36]. The growth pattern is considered to be anisotropic due to size increase of the NPs. Furthermore, in the mechanism, Ag (I) got reduced first because of its higher positive electrochemical potential than nickel which led to the formation of silver core [37].

### 3.4. Antimicrobial Activity

#### 3.4.1. Antimicrobial Assay

**Figure 7:**
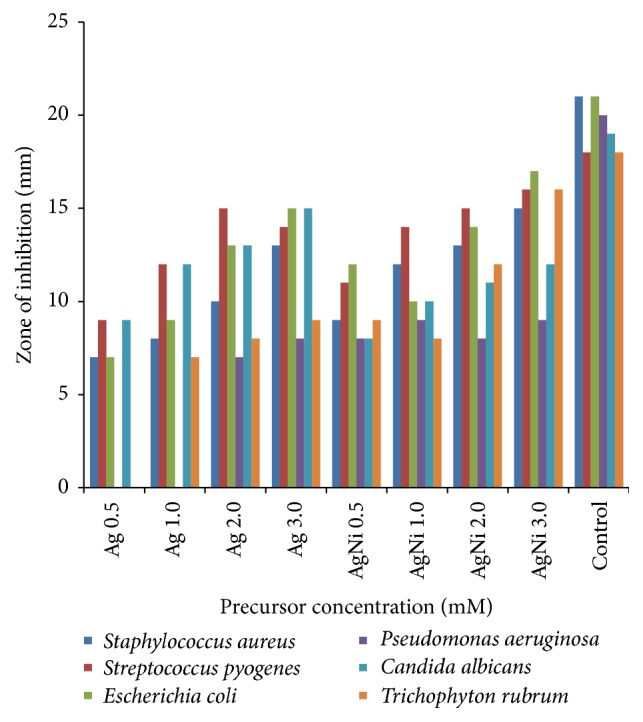
Comparison of inhibition zones between Ag NPs and Ag-Ni bimetallic nanoparticles synthesized using* C. indica* leaf extract.

Activity of the biosynthesized Ag NPs and Ag/Ni bimetallic nanoparticles based on size of zones of inhibition in millimetre (mm) is shown in Figure 7. Agar diffusion test revealed that the prepared nanoparticles possessed both antibacterial and antifungal properties. The screened nanoparticles exhibited higher activities on all the test organisms at higher concentration of 3.0 mM except* P. aeruginosa* in which low activity was recorded. Sensitivity testing of organisms (Agar diffusion test) in triplicate showed zones of inhibition. Mean zone of inhibition diameter (mm) ± standard deviation is shown in Table 2. One-way analysis of variance (ANOVA) using SPSS statistical tool indicated that growth inhibition by the nanoparticles was significant at *P* < 0.05, *F*-value 34.06 (Table 3).

Zones of inhibition recorded in agar well diffusion test led to the conduction of minimum inhibitory concentration (MIC), minimum bactericidal concentration (MBC) and minimum fungicidal concentration (MFC) tests. Results of MIC, MBC, and MFC tests are presented in Table 3. Interestingly, all prepared nanoparticles showed concentration-dependent inhibitory effects on the* in vitro* antimicrobial assay [38]. Highest activity of Ag NPs was on* E. coli* and* C. albicans* with MIC value of 12.5 mg/mL and 2.5 mg/mL (same value for MBC and MFC), followed by* S. aureus* and* S. pyogenes *(12.5 mg/mL value of MIC and MBC). The activity was least on* T. rubrum *(50 mg/mL MIC, 100 mg/mL MFC). However, no detectable activity was found against* P. aeruginosa*. Higher activity was observed in Ag/Ni nanoparticles against* S. pyogenes *with MIC value of 6.25 mg/mL and 12.5 mg/mL MBC.

In hybrid Ag/Ni nanoparticles, analysis of variance (ANOVA) using SPSS statistical tool indicated no significant difference in the concentrations as *P* > 0.05, yet possessing better activity than its corresponding Ag NPs which was observed at different levels in the test organisms. Moreover, these nanoparticles could not hinder the growth of* P. aeruginosa*. None of the as-synthesized nanoparticles was able to compete with ciprofloxacin and fluconazole (standards) in terms of activity.* S. aureus, S. pyogenes,* and* E. coli* showed similar behaviour relative to MIC and MBC, while* P. aeruginosa* was highly resistant. The metallic nanoparticles were more active on* C. albicans* than* T. rubrum* possibly because they were able to penetrate the thin peptidoglycan layer of the fungus with the outer membrane composed of phospholipids and lipopolysaccharides (LPS) of the* E. coli *(complex gram-negative bacterium). Not only were the bionanoparticles considered to have passed through thicker peptidoglycan cell wall layer which is accountable for rigidity and low activity in gram-positive bacteria, as detected in the MIC test carried out on* S. aureus* and* S. pyogenes* [39], but, according to Marini et al., the observed growth inhibition in bacteria can also be related to the reaction of thiol groups present in bacteria protein with the release of Ag^+^ which slowed down or changed the replication of DNA [40].

From the above, there is a need to affirm if the penetrations of the nanoparticle through the microbial samples influence its experimental result. Tan et al. [41] did the characterization of nanoparticle dispersion through red blood cell using the nanoparticle model. Through experimentation, the nanoparticle dispersion model was given as

(1) $$\begin{array}{c} D_{r}=\frac{D-D_{0}}{{d_{layer}}^{2}\eta }. \end{array}$$

*D*_*r*_ is the dimensionless dispersion rate, *D* is the dispersion rate, *D*_0_ is the dispersion tendency, *d* is the cell/layer, and *η* is the shear rate. Tan et al. [41] worked with the shear rate below 40 s^−1^ and above 200 s^−1^.

Kleinstreuer and Xu [42] hence gave *D*_0_ for metallic nanoparticles penetration through the thin peptidoglycan layer as

(2) $$\begin{array}{c} D_{0}=\frac{k_{B}T}{3\pi \mu_{bf}d_{p}}, \end{array}$$

where *k*_*B*_ is the Stefan Boltzmann constant, *T* is the local temperature, *μ*_bf_ is the base fluid viscosity, and *d*_*p*_ is the particle diameter. From past experimentation, Benakashani et al. [43] found average particle size of Ag NPs using the Debye-Scherrer equation:

(3) $$\begin{array}{c} d_{nano}=\frac{K\lambda }{\beta \cos\theta }, \end{array}$$

where *d* is the size of the Ag NPs which is about 20 nm, *K* is the Scherrer constant that ranges between 0.9 and 1, *λ* is the wavelength of the X-ray source, in this case it ranges between 280 and 780 nm, *β* is the full width at the half maximum of the diffraction peak, and *θ* is Bragg's angle.

The basic condition for the metallic nanoparticle to go through the polymer matrix of the thin peptidoglycan layer is *d*_nano_ < *d*_layer_. Hence,

(4) $$\begin{array}{c} d_{layer}=d_{nano}+d_{o}. \end{array}$$

The dimensionless dispersion of the metallic nanoparticle through the peptidoglycan layer is given as

(5) Dr=DK2λ2/β2cos2θ+2Kλd0/βcos⁡θ+d02η−1K2λ2/β2cos2θ+2Kλd0/βcos⁡θ+d02η·kBT3πμbfdp,

where

(6) $$\begin{array}{c} D=\left\{\begin{array}{cc} \frac{1}{4}\cos\left(\frac{\pi d_{p}}{4}\right) & d_{p}<10^{-9}\\ 0 & otherwise. \end{array}\right. \end{array}$$

When *D* = 0, then the dimensionless dispersion becomes

(7) Dr=1K2λ2/β2cos2θ+2Kλd0/βcos⁡θ+d02η·kBT3πμbfdp.

**Figure 8:**
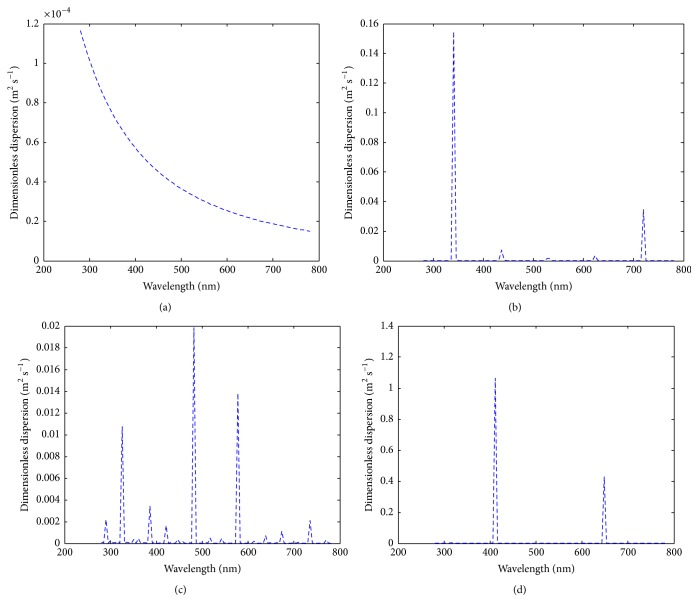
The dimensionless dispersion of the metallic nanoparticle under varying conditions (a) when Bragg's angle of the nanoparticle is strictly 45°; (b) when Bragg's angle of the nanoparticle ranges between −30° and 30°; (c) when Bragg's angle of the nanoparticle ranges between −45° and 45°; (d) when Bragg's angle of the nanoparticle ranges between −60° and 60°.

**Figure 9:**
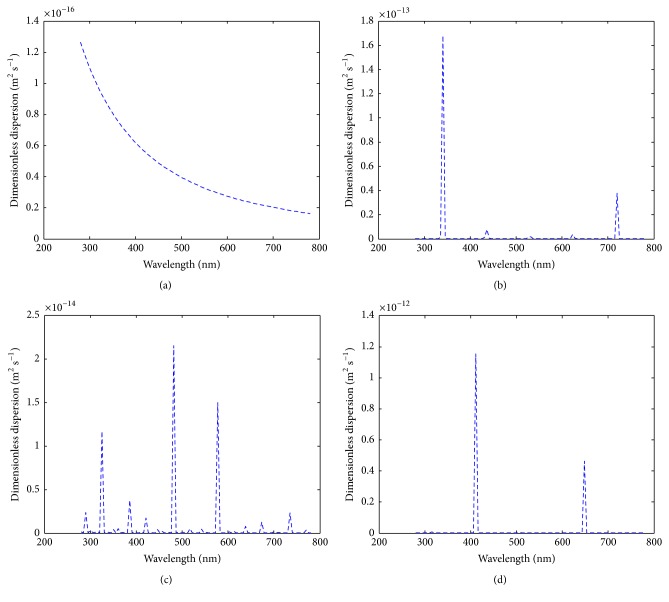
The dimensionless dispersion of the metallic nanoparticle under varying conditions at diameter greater than 10^−9^ nm (a) when Bragg's angle of the nanoparticle is strictly 45°; (b) when Bragg's angle of the nanoparticle ranges between −30° and 30°; (c) when Bragg's angle of the nanoparticle ranges between −45° and 45°; (d) when Bragg's angle of the nanoparticle ranges between −60° and 60°.

Hence, the illustration of the two cases is shown in Figures 8(a)–8(d) and 9(a)–9(d).

The first case (i.e., (5)) was when the diameter of the metallic particle is equal to or less than 10^−9^ m. At a constant Bragg's angle of 45°, the feature of the dispersion/wavelength trend (Figure 8(a)) is the same as results shown in Figures 2 and 3. This is the first evidence that the penetration of metallic nanoparticles through the thin peptidoglycan layer does not necessarily influence the efficiency of its constituents. Then in a case when the sizes of the nanoparticle are heterogeneous, we assumed Bragg's angle of the nanoparticle ranges between −30° and 30° (Figure 8(b)). Five peaks appeared with the maximum peak at wavelength 340 nm. The significance of this result can be seen in its effect in Figures 2(a)–2(d). A further analysis was conducted to see the features of the dimensionless dispersion of the nanoparticles when Bragg's angle of the nanoparticle ranges between −45° and 45° (Figure 8(c)). Fifteen peaks were observed showing the response or sensitivity of Bragg's angle to nanoparticle transport within multilayers. Then Bragg's angle when the nanoparticle range is between −60° and 60° was considered (Figure 8(d)). Only two peaks were observed. Hence, the penetration of nanoparticle does not depict the activeness of the microbial samples in all cases which largely depend on the nature and pore size of the peptidoglycan layer.

The second case (i.e., (7)) was when the diameter of the metallic particle is greater than 10^−9^ m (Figures 9(a)–9(d)). It was observed that the features of Figures 8 and 9 were the same. However, the dimensionless dispersion is very low.

## 4. Conclusion

Rapid, facile, and environmental-friendly syntheses of monometallic Ag NPs and Ag/Ni bimetallic nanoparticles using* Canna indica* leaf extract were successful via reduction of AgNO_3_ and Ni(NO_3_)_2_·6H_2_O metal precursors. No doubt, the leaf extract acted as the reducing/capping agent. The metallic nanoparticles were characterized for their optical and morphological properties. Nucleation and onset growth which were considered to be by diffusion control and Ostwald's ripening commenced as early as 5 minutes. Ag NPs had maximum absorption in the visible region with absorption wavelength of 416 nm. However, presence of nickel (Ni) in the hybrid synthesis led to a red shift in the absorbance wavelength from 416 to 421 nm after 30 minutes of the reaction. TEM image revealed quasi-spherical shapes of average diameter 9.10 ± 1.12 nm in Ag NPs while micrograph of the bimetallic Ag/Ni cluster revealed different shapes: cube with truncated/irregular edges plausibly due to the effect of Ostwald ripening and multiply twinned hybrid after 30 minutes of the reaction with a mean particle size of 9.86 ± 2.37 nm. Structural elucidation from the TEM image also revealed formation of core-shell Ag/Ni nanoparticles. The denser silver particles were distinctly visible in the TEM image. The Ag nanoparticles appeared as a dark core with Ni particles appearing less dark on the surface (Figure 2(d)). EDX analysis showing elemental compositions of the nanoparticles indicated that the nanohybrid was silver enriched with organic capping due to the composition of carbon content which originated from plant extract. Furthermore, Ag/Ni bimetallic nanocluster exhibited better antimicrobial activity against the test pathogens than its corresponding monometallic Ag NPs. Hence, from the findings, Ag/Ni NPs are potential antibacterial agents against* E. coli* and possible antifungal agents against* C. albicans*. Possible antibacterial drugs against* S. pyogenes* and* E. coli* can be designed using Ag-Ni nanohybrid, based on their strong inhibition activities observed. The observed enhanced SPR in the nanoclusters is noted for applications in optical materials, also as good absorbers of visible light absorber and scatters.

## Figures and Tables

**Table 1:** Phytochemical analysis of *Canna indica *leaf extract.

Phytochemical										
	Proteins	Carbohydrates	Phenols	Tannins	Flavonoids	Saponins	Glycoside	Steroids	Terpenoids	Alkaloids
Aqueous extract	−	−	−	−	−	−	++	−	+	+++
Methanolic extract	−	−	−	+++	++	+	++	−	++	+++

Weak presence +; strong presence ++; stronger presence +++; absent −.

**Table 2:** Sensitivity testing of organisms with standard deviation in zones of inhibition (agar diffusion test).

NPs	Organisms/mean zone diameter (mm) ± SD					
	*Staphylococcus aureus*	*Streptococcus pyogenes*	*Escherichia* *coli*	*Pseudomonas* *aeruginosa*	*Candida* *albicans*	*Trichophyton* *rubrum*
Ag 0.5	7 ± 0.2	9 ± 0.4	7 ± 0.1	Nil	9 ± 0.2	Nil
Ag 1.0	8 ± 0.5	12 ± 0.8	9 ± 0.2	Nil	12 ± 0.3	7 ± 0.2
Ag 2.0	10 ± 0.4	15 ± 0.5	13 ± 0.4	7 ± 0.2	13 ± 0.4	8 ± 0.1
Ag 3.0	13 ± 1	14 ± 0.2	15 ± 0.3	8 ± 0.1	15 ± 0.2	9 ± 0.1
*Stat*	*P > 0.05*	*P > 0.05*	*P > 0.05*	*P > 0.05*	*P > 0.05*	*P > 0.05*
Ag-Ni 0.5	9 ± 0.2	11 ± 0.4	12 ± 0.5	8 ± 0.3	8 ± 0.1	9 ± 0.2
Ag-Ni 1.0	12 ± 0.3	14 ± 0.6	10 ± 0.2	9 ± 0.2	10 ± 0.2	8 ± 0.1
Ag-Ni 2.0	13 ± 0.1	15 ± 0.2	14 ± 0.4	8 ± 0.1	11 ± 0.2	12 ± 0.3
Ag-Ni 3.0	15 ± 0.4	16 ± 0.6	17 ± 0.6	9 ± 0.1	12 ± 0.1	16 ± 0.2
Control	21 ± 0.8	18 ± 0.3	21 ± 0.2	20 ± 0.4	19 ± 0.6	18 ± 0.3
Stat	*P* > 0.05	*P* > 0.05	*P* > 0.05	*P* > 0.05	*P* > 0.05	*P* > 0.05

Control: ciprofloxacin (bacteria) and fluconazole (fungi); mean zone inhibition (mm) ± standard deviation of triplicate measurements. Ag = silver nanoparticles of specified precursor concentration using *C. indica* leaf extract; Ag/Ni = silver-nickel bimetallic nanoparticles of specified precursor concentration using *C. indica* leaf extract.

**Table 3:** Minimum inhibitory concentration (MIC), minimum bactericidal concentration (MBC), and minimum fungicidal concentration (MFC).

Nanoparticles	Organisms/MIC, MBC & MFC (mg/mL)					
	*Staphylococcus aureus*	*Streptococcus pyogenes*	*Escherichia coli*	*Pseudomonas aeruginosa*	*Candida albicans*	*Trichophyton rubrum*
	MIC, MBC	MIC, MBC	MIC, MBC	MIC, MBC	MIC, MFC	MIC, MFC
Ag 0.5	100, 100	50, 100	100, 100	100, 100	50, 50	100, 100
Ag 1.0	100, 100	25, 50	50, 100	100, 100	25, 25	100, 100
Ag 2.0	50, 100	12.5, 25	12.5, 25	100, 100	12.5, 25	100, 100
Ag 3.0	12.5, 25	12.5, 25	12.5, 12.5	100, 100	12.5, 12.5	50, 100
*Statistics*	*P < 0.05*	*P < 0.05*	*P < 0.05*	*P < 0.05*	*P < 0.05*	*P < 0.05*
Ag/Ni 0.5	50, 100	25, 50	12.5, 25	100, 100	50, 100	50, 100
Ag/Ni 1.0	12.5, 25	12.5, 12.5	25, 50	50, 100	25, 50	100, 100
Ag/Ni 2.0	12.5, 25	12.5, 12.5	12.5, 12.5	100, 100	12.5, 25	12.5, 12.5
Ag/Ni 3.0	12.5, 12.5	6.25, 12.5	6.25, 12.5	100, 100	12.5, 25	12.5, 25
*Statistics*	*P < 0.05 *	*P < 0.05 *	*P < 0.05 *	*P < 0.05 *	*P < 0.05 *	*P < 0.05 *
Control	*3.13*	*6.25*	*6.25*	*6.25*	*6.25*	*6.25*
